# EEG alpha/beta features as a biomarker for quantifying pain in patients with lumbar disk herniation

**DOI:** 10.3389/fnins.2025.1507245

**Published:** 2025-02-11

**Authors:** Rumei Li, Wanqi Shao, Shumei Zhao, Lingli Wang, Chao Yu, Lanying Liu, Kuiying Yin

**Affiliations:** ^1^Nanjing Research Institute of Electronics Technology, Nanjing, China; ^2^National Key Laboratory of Radar Detection and Sensing, Nanjing, China; ^3^Affiliated Hospital of Nanjing University of Chinese Medicine, Nanjing, China

**Keywords:** electroencephalography, biomarker, power ratio, oscillations, lumbar disk herniation, pain

## Abstract

**Introduction:**

An objective and precise pain evaluation is of significant clinical value, and electroencephalography as a non-invasive physiological signal has been demonstrated to correlate with subjective pain perception. This study aimed to analyze the EEG changes in patients with lumbar disk herniation (LDH) under traditional Chinese medicine small needle knife and to further explore the feasibility of EEG as an indicator of pain assessment in patients with LDH.

**Methods:**

This study conducted resting-state electroencephalography on 20 patients with LDH before and after treatment and on 20 healthy controls, respectively. Following the spectral analysis of the EEG signals with continuous wavelet transform, power ratios were extracted for four frequency bands (θ, α, β and γ). Significance tests were conducted within the LDH group and between the LDH and healthy controls, as well as correlation analyses of EEG characteristics with pain scales in four regions of interest.

**Results:**

A significant reduction in subjective pain intensity was observed after small needle knife, with a 32.86 and 38.41% reduction in the Visual Analog Scale (VAS) and modified Japanese Orthopedic Association (mJOA) scores, respectively. Alpha accounted for a significantly higher of the four regions, while theta in the frontal, occipital and beta in the central were significantly lower. HC had fewer EEG oscillations in the theta band compared to LDH. The constructed alpha/beta features demonstrated a significant negative correlation with VAS in the frontal (*R* = −0.361, *P* = 0.022) and parietal (*R* = −0.341, *P* = 0.031), as well as with mJOA in the frontal (*R* = −0.416, *P* = 0.007), central (*R* = −0.438, *P* = 0.004), and parietal (*R* = −0.390, *P* = 0.013) regions.

**Conclusion:**

EEG power ratios showed significantly different results in LDH groups, and between patients and HC. The alpha/beta features of the frontal and parietal constructed in this study showed correlations with subjective pain scores and might serve as a biomarker of pain status in the short term in LDH.

## 1 Introduction

Lumbar disk herniation (LDH) is a prevalent and frequently occurring condition in spinal surgery, and is defined as the displacement of the nucleus pulposus of the intervertebral disk through the annulus fibrosus beyond the normal edge of the dis. It is mainly caused by lumbar intervertebral disk degeneration, nucleus pulposus protrusion, spinal nerve root compression and other comprehensive factors (Kreiner et al., 2014). The characteristic symptom of LDH is lower back pain caused by nerve compression, and the pain may radiate to the legs and feet (Knezevic et al., 2021). For Chinese people, the prevalence of LDH in the last 20 years is 6%, and the prevalence tends to increase with age and generation, reaching 11% in people over 60 years old (Xu B. et al., 2023). While some patients with LDH recover without treatment, others experience intolerable and persistent pain (Weber, 1983). LDH pain is generally treated with nonpharmacological treatments that include massage, traction, physical therapy, and acupuncture to phase out the pain (Schoenfeld and Weiner, 2010). Some patients also use a combination of non-pharmacologic and pharmacologic treatments but do not address the underlying cause of pain (Knezevic et al., 2017). Surgery may resolve the root cause of LDH, but potential complications are difficult to avoid (Liu et al., 2023). Non-surgical, minimally invasive treatments that provide sustained pain relief are needed.

The small needle knife is a traditional Chinese medicine intervention widely used in the treatment of chronic pain (Ma et al., 2010; Rumei Li et al., 2016). The knife is a metal needle with a diameter of 0.4 to 1 mm, the metal needle has a wider tip than clinical filiform needles (Zhang D. et al., 2019). The wider tip allows the knife to stimulate or block nerve conduction by cutting and releasing the fascia, relieving pain by stripping away harmful tissue fibers (Rabenstein et al., 1997). Small needle knife is a commonly used method in the treatment of Knee osteoarthritis (Xie et al., 2020), Myofascial pain syndrome (Zhang D. et al., 2019), and has good clinical effects on relieving low back pain in patients with LDH, with quick efficacy and less traumatization to patients, but the neural mechanism of analgesia still needs to be further explored (Song et al., 2022).

Pain is influenced by various factors, including tissue damage, sensation, emotion, and cognition, and is considered a subjective individual experience (Davis et al., 2017; Raja et al., 2020). In clinical research, the measurement and assessment of pain are realized through self-reporting, relying on numerical rating scales or visual analog scales, and the assessment process depends on the guidance of experienced physicians (Puljak et al., 2021). Pain is a multidimensional and subjective experience, which makes the assessment of pain more complex, and patients’ self-reports are conducted according to how they feel different aspects of pain, such as intensity, duration, and emotional impact (Scher et al., 2020). And the way the scale is reported is somewhat dependent on the ability of the patient to communicate and express themselves (Rowbotham et al., 2014). Therefore, it is crucial to develop a non-invasive objective measurement method for pain to monitor the evolution of pain levels to assist doctors in making better diagnoses, treatments, and patient management (Nir et al., 2010; Puljak et al., 2021). Objective measurement methods should be sensitive and accurate and free from subjective influences of both the patient and the evaluator (Shankar and Abudhahir, 2021). It can also address the issue of assessing pain in special patient populations who cannot communicate due to limitations in communication and cognition (Kaiser et al., 2020).

Electroencephalography (EEG) is a non-invasive neuromonitoring technology that reflects the real-time dynamic activity of the brain by measuring postsynaptic potentials (Gomez-Pilar et al., 2020). It is characterized by its low cost and ease of use, offering immense potential in diagnosing pain and monitoring the progression of conditions. In EEG analysis, signals are typically decomposed into five different frequency bands: delta (0.1–3 Hz), theta (4–7 Hz), alpha (8–13 Hz), beta (14–30 Hz), and gamma (30–50 Hz). In general, delta represents deep sleep or unconsciousness; theta is associated with primal emotions and deep feelings, and alpha indicates a state of conscious relaxation. Beta increases when the brain is engaged in cognitive reasoning and computational tasks. Gamma mainly reflects more advanced mental activities (Gross, 2014).

Pain pulses in peripheral sensory neurons are transmitted to the spinal cord through the dorsal root ganglia. Projections are sent to the thalamus via spinal cord neurons and then arrive at the cerebral cortex (Ringkamp et al., 2018). EEG has been employed in numerous studies to characterize pain sensation, with findings indicating a correlation between theta, alpha, beta, and gamma oscillations in the brain and pain perception. Colon et al. (2017) discovered that periodic heat stimulation induces periodic modulation of EEG oscillation amplitudes in the theta, alpha, and beta bands. In patients with chronic low back pain, there is a positive correlation between the intensity of persistent pain and beta and gamma oscillations in the frontal, with chronic pain patients exhibiting more robust theta oscillations in their EEG (Ye et al., 2019). Nickel et al. (2017) discovered that the theta, alpha, beta, and gamma frequency bands all correlate with pain intensity, with pain responses to different stimulus intensities being more sensitive in the sensory cortex. Current research indicates an association between pain and EEG, with this association present across different frequency bands such as theta, alpha, beta, and gamma. However, the trends of EEG fluctuations within each band are inconsistent across studies. Some studies suggest that pain stimulation increases theta rhythm (Dufort Rouleau et al., 2015; Schulz et al., 2015; Taesler and Rose, 2016), while others report a decrease in theta activity (Gram et al., 2015; Bunk et al., 2018). Common research posits a negative correlation between alpha in the parietal-occipital region and pain (Chang et al., 2001; Gram et al., 2015; Schulz et al., 2015; Bunk et al., 2018; Feng et al., 2021), although some studies have reported opposite findings (Schulz et al., 2015; Huishi Zhang et al., 2016). In most studies, there is a positive correlation between the power in the beta and gamma frequency bands and pain intensity (Gram et al., 2017; Chouchou et al., 2021); however, some research suggests that pain may suppress the beta rhythm (Bunk et al., 2018).

In the last decade or so, in work on the relationship between EEG and pain, researchers have evoked pain employing electro-laser or temperature stimulation (Schulz et al., 2015; Bunk et al., 2018; Wang et al., 2023). Other researchers have focused on clinical pain, finding EEG-based specificity by analyzing the relationship between pain assessment scale scores and patients’ EEG characteristics (Kumar et al., 2015; Feng et al., 2021). Still other studies have compared differences in EEG specificity between pain patients and healthy individuals (Di Pietro et al., 2018; Zhou et al., 2018). In induced pain research, most studies have used healthy individuals as subjects (Babiloni et al., 2002; Schulz et al., 2015; Bunk et al., 2018; Wang et al., 2023). By controlling the intensity of different stimulation approaches the corresponding pain level is represented, for example the level of stimulation temperature is used to represent the pain intensity in heat stimulation (Huber et al., 2006). This approach leads to a quantitative description of pain levels and facilitates the construction of a relationship between stimulus intensity (pain level) and EEG (Zis et al., 2022). However, stimulus-induced pain responses in healthy individuals may have different neural processing than pain in clinical patients (de Vries et al., 2013; Mouraux and Iannetti, 2018; Reckziegel et al., 2019). The conclusions drawn from the three types of studies may not be applicable to each other, and therefore, these current studies have not yet produced an EEG biomarker for all pain perception (Mussigmann et al., 2022; Zebhauser et al., 2023). It would be more meaningful to further refine the classification for different pain types and pain sources, as well as for groups of subjects, and to conduct studies within specific groups (Pinheiro et al., 2016). In this study, we addressed chronic low back pain due to LDH and found an objective neurophysiological parameter to quantitatively measure and quantify pain through the different rhythmic characteristics of the EEG signals of the patients in the closed-eye state.

In this research, we extracted signal power characteristics across various brain rhythms. We performed comparative analyses between LDH group before and after small needle knife treatment, as well as between the LDH group and healthy subjects. Comparison of EEG patterns in different pain states was used to verify the differences in the pattern of EEG changes within and between groups. Ultimately, it is hoped that correlation analysis between EEG features and subjective pain scales will be used to find an EEG biomarker for LDH patients.

## 2 Materials and methods

### 2.1 Subjects

Patients with LDH attending Jiangsu Province Hospital of Chinese Medicine during 2023 were selected, and 20 patients (12 males and 8 females) fulfilled the diagnostic and inclusion criteria after careful clinical assessment by clinicians. Subjects included in this study were asked to read the study protocol carefully and sign an informed consent form to join the experiment. The mean age of the subjects in the patient group was 46.1 years (age range 27–66 years); the disease duration ranged from 1 month to 10 years, with a mean duration of 3.8 years. All patients experienced pain for more than 3 months, except for one patient who had pain for 1 month. In addition, 20 healthy individuals with a similar sex ratio and age distribution as the patient group were recruited as healthy controls (HC). Each subject provided informed written consent before each experiment. The study was conducted by the guidelines of the Declaration of Helsinki. The experimental protocol was approved by the Affiliated Hospital of Nanjing University of Chinese Medicine (2023NL-272-02). In the follow-up experiments, some subjects did not cooperate reasonably in introducing excessive noise during EEG acquisition, and the data of one healthy control subject were not analyzed in the end.

### 2.2 EEG recording

**FIGURE 1:**
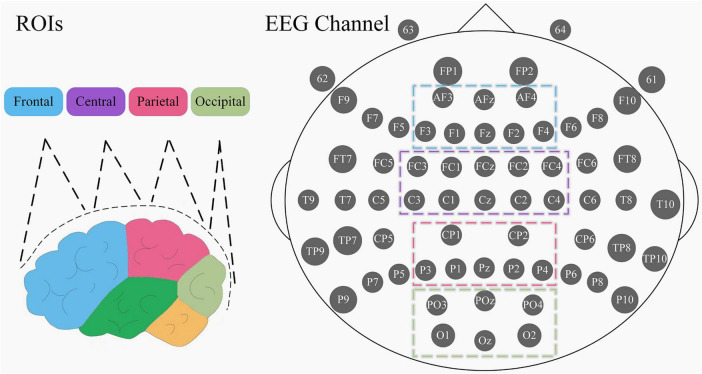
Division of the 4 ROIs and electrode names of the EGI EEG device. The left picture is the side view, different color blocks represent different brain regions. Electrode positions are in top view, with each gray circle representing an acquisition electrode, the large ellipse on the periphery representing the human brain cranium, the small ellipses on either side representing the ears, and the sharp corner above the tip of the nose position.

EEG was recorded by 64-channel Geodesic EEG Systems from EGI and the accompanying Net Acquisition software. The device uses a saline electrode cap with 64 active electrodes and 1 default reference electrode (Cz), and the electrode positions are arranged using the international 10–20 standard. The names and locations of the electrodes are shown in Figure 1. Data were recorded at a sampling frequency of 1,000 Hz with 0.01–100 Hz bandpass filtering set in the acquisition software, and the impedance of all electrodes was kept below 50 KΩ.

### 2.3 Small needle knife intervention

The patient was lying prone on the bed. The doctor searches for the pain point in the patient’s lumbar multifidus muscle distribution by repeated touching, and then marks and locally sterilizes the skin. A 0.60 mm × 50 mm disposable needle knife was selected to be pushed vertically into the marked pain point for incision, stripping, release and cutting. The direction of the cutting edge of the blade was kept parallel to the muscle fibers, nerves and blood vessels during the operation to prevent patient injury (Zhu et al., 2020). The treatment time for each patient is 5–10 min, which is determined by the doctor according to the patient’s condition.

### 2.4 Experimental procedure

**FIGURE 2:**
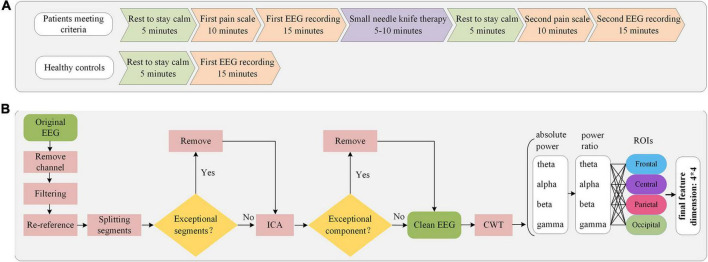
Flowchart of experimental procedure and EEG data analysis. **(A)** Experimental procedures and sequence of data collection in patients and HC. **(B)** Processing sequence of EEG preprocessing and feature extraction.

Before the experiment, LDH patients were asked to rest inside a quiet, temperature-controlled room for 5 min to remain calm. Under the guidance of an experienced physician, subjects are familiar with the pain scale and the experimental procedure. The Visual Analog Scale (VAS, with a score of 0 for “no pain sensation” and 10 for “the worst imaginable pain”) (Shafshak and Elnemr, 2021) and modified Japanese Orthopedic Association Scores (mJOA, 0–30, with larger scores indicating higher pain) (Wang et al., 2022) were used to assess the pain intensity of the patients. Subsequently, the subjects were ensured to be comfortable sitting and positioned, and resting-state EEG data were collected for 15 min with the subjects’ eyes closed and relaxed. The subjects were then subjected to small needle knife therapy. The pain scale and 15-min closed-eye resting EEG acquisition were performed again after another 5-min rest to calm after the treatment. HC underwent only one closed-eye EEG acquisition with the same duration and acquisition environment as LDH group. The experimental flow and data acquisition sequence for both groups are shown in Figure 2A.

### 2.5 EEG preprocessing

The data were subjected to preprocessing using the EEGLAB toolbox (Delorme and Makeig, 2004). The electrodes T9, T10, E61, E62, E63 and E64 were not used, and the corresponding EEG data were excluded. The steps for EEG preprocessing were referenced from previous work (Zhao et al., 2023). A bandpass filter was employed, with a passband ranging from 0.5 to 49 Hz, to eliminate industrial frequency interference and low-frequency noise. The data were re-referenced using global averaging. Due to the special characteristics of LDH, it is difficult for patients to avoid postural adjustments due to pain during data acquisition. This can cause phase abnormalities in the EEG data. To address the issue of abnormal EEG data, a sliding window of 10 s in length, without repetition, was employed to segment the data following re-referencing. The data segments exhibiting aberrant characteristics were subsequently removed. Independent component analysis (Makeig et al., 1995) was then performed and possible eye movement artifact components were removed . The final data dimension obtained for each subject was 59*10000*n, where n represents the number of segments.

### 2.6 EEG feature extraction

The time-frequency analysis of the preprocessed signal is performed using the continuous wavelet transform (CWT) to decompose the original signal under five commonly used frequency bands. CWT can preserve the time and frequency domain features of the original signal and is widely used in EEG analysis (Zhang D. et al., 2019). For the signal *x*(*t*), the continuous wavelet transform can be described by the following expression (Jadhav et al., 2020):


$$\begin{array}{c} W\,T=\left\langle x\,\left(t\right),\psi_{a,b}\,\left(t\right)\right\rangle \end{array}$$

$$=\frac{1}{\sqrt{a}}\int_{-\infty }^{+\infty }x\left(t\right)\Psi^{*}\left(\frac{t-b}{a}\right)dt,\left(a>0\right)$$

and *a* is the scaling factor (dilation or compression), and *b* is the translation factor (time shift). ψ* is the complex conjugate form of the wavelet function ψ. The essence of the continuous wavelet transform is the convolution of the original signal with the wavelet function. As *a* and *b* vary, a series of wavelet functions can be obtained from the wavelet mother function:


$$\psi_{a,b}\,\left(t\right)=\frac{1}{\sqrt{a}}\,\psi \,\left(\frac{t-b}{a}\right)$$

The pseudo-frequency *F*_*a*_ of the wavelet coefficients *WT* is expressed as *F*_*a*_ = *F*_*c*_/(*a* × △), where *F_c_* is the center frequency of the mother wavelet and △ is the sampling period. The corresponding wavelet coefficients were then averaged according to the upper and lower limits of the commonly used frequency bands to obtain the data under the five frequency bands. The power spectral density is then calculated using Welch’s method and the average power values under each frequency band are further calculated using the integral estimation method of rectangular approximation.

In the process of EEG signal acquisition, there is a propensity for the introduction of low-frequency noise. Previous works pertaining to EEG analyses in pain-related studies have indicated a significant correlation between the initiation of pain and high-frequency oscillations. Consequently, to mitigate potential noise-related issues, the present investigation exclusively examines the theta, alpha, beta, and gamma frequency bands (Hu et al., 2013; Schulz et al., 2015; Baumgarten et al., 2016). Furthermore, given the variability in EEG characteristics across subjects, employing absolute power values may engender inaccuracies. Therefore, the utilization of power ratios as the definitive feature set is warranted in this context. With reference to previous studies (Oken et al., 2014; Hu and Lodewijks, 2020; Usama et al., 2022), four horizontally arranged regions of interest (ROIs) were selected: frontal (FP1, FP2, AFz, AF3, AF4, Fz, F1, F2, F3, F4), central (FCz, FC1, FC2, FC3, FC4, Cz, C1, C2, C3, C4), parietal (CPz, CP1, CP2, CP3, CP4, Pz, P1, P2, P3, P4) and occipital (POz, PO3, PO4, Oz, O1, O2) (Figure 1). Ultimately, we calculated the power ratio of the four frequency bands of the four ROIs in each EEG segment of each subject and took the mean value between segments as the final feature value. The flowchart of EEG preprocessing and feature extraction is shown in Figure 2B.

### 2.7 Statistical analysis

Statistical computations were performed using the MATLAB software environment. In the evaluation of correlation, Spearman’s rank correlation coefficient was employed. Spearman’s coefficient is sensitive only to the monotonic relationship between variables, is indifferent to the actual values, and is resilient to the presence of outliers. Data obtained before and after treatment were concurrently analyzed when calculating the correlation coefficients. In the within-group test for LDH, the sequence of differences in power ratios pre- and post-treatment was first subjected to the Lilliefors test to ascertain normality. A paired-sample *t*-test was applied for those sequences exhibiting normal distribution; for those that did not, the non-parametric Wilcoxon signed-rank test was utilized. The Wilcoxon rank-sum test was employed for the comparative analysis of variances between LDH group and HC. All statistical significance set at a threshold of *P* < 0.05.

### 2.8 Instruments and assessment of outcomes

The main goal of this study was to explore the pattern of EEG changes during pain alterations in patients with LDH in order to find an EEG biomarker capable of assessing pain. We performed significance tests of power ratio before and after treatment and a correlation analysis of power ratio with scale scores within the LDH group, all of which were performed independently in each frequency band and within each brain region. Comparisons between HC and LDH groups were also performed to validate between- and within-group differences in the relationship between EEG and pain and to provide limits to the range of applicability of the biomarkers. Changes in scales, as well as overall changes in EEG status, will also be included in the results. After finding individual EEG frequency bands that were positively or negatively correlated with pain based on the results of the between- and within-group analyses, we sought to strengthen the correlation of EEG features with supervisor pain by constructing ratios between the bands and thus constructed three possible pairings of alpha/beta, alpha/theta, and alpha/(theta+beta). The three constructed features were similarly analyzed for correlation with subjective pain scores.

## 3 Results

### 3.1 Differences within LDH group

**FIGURE 3:**
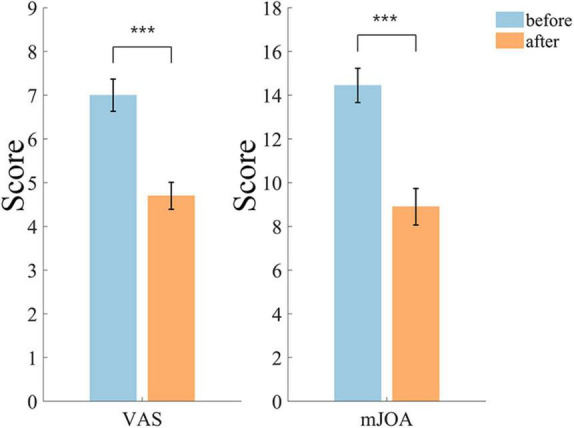
Differences in VAS and mJOA scores before and after treatment. The level of significant difference is indicated by the use of * in the graphs, *p < 0.05, **p < 0.01,***p < 0.001, and the same significance is used in subsequent graphs.

The difference of VAS and mJOA scores is shown in Figure 3, the VAS scores were reduced by 32.86% (*p* = 0.000078) after treatment, and the mean value on 20 subjects decreased from 7.00 to 4.70. mJOA scores were significantly reduced by 38.41% (*p* = 0.000084), and the mean value decreased from 14.45 to 8.90.

**FIGURE 4:**
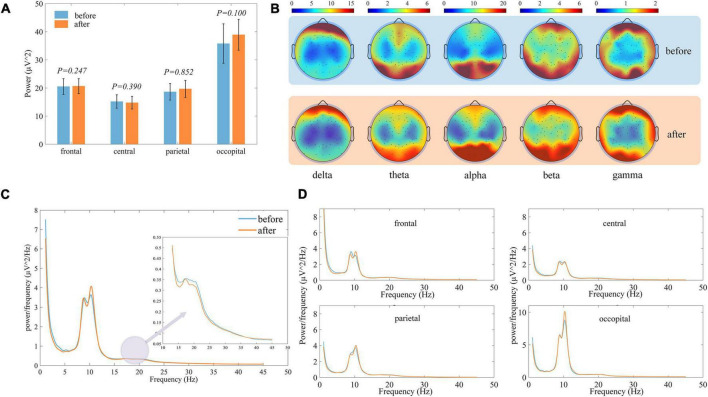
Comparison of EEG characteristics of all subjects before and after treatment, in each figure blue represents before treatment and orange represents after treatment. **(A)** Histogram of the distribution of the total EEG power of the subjects within different brain regions. **(B)** Topographic map of the distribution of the EEG power in different frequency bands. **(C)** Comparison of the power spectral density curves averaged over the whole brain. **(D)** Comparison of the power spectral density curves averaged over the various brain regions.

Figure 4A shows the distribution of total EEG power within the different brain regions, and the large variance between subjects shows subject-specificity. When the total EEG power before and after treatment was tested for differences, there were no significant differences within each ROI, indicating that the total power did not change significantly before and after treatment. Therefore, in the subsequent analysis we focus more on the relative ratio of frequency bands. A more detailed topographic power distribution of the brain for each sampled electrode is shown in Figure 4B. Figure 4C shows the average power spectral density curves of the whole brain before and after treatment for all LDH group subjects, alpha is the dominant frequency band. After the small needle knife, the average power distribution changed only slightly, mainly in the theta band. In contrast, the oscillation in the alpha band was strengthened, the beta decreased after the treatment, and the overall average power in gamma did not change much. In the power spectral density curves of the individual ROI shown in Figure 4D, the trend of change was similar to that of the whole brain.

**FIGURE 5:**
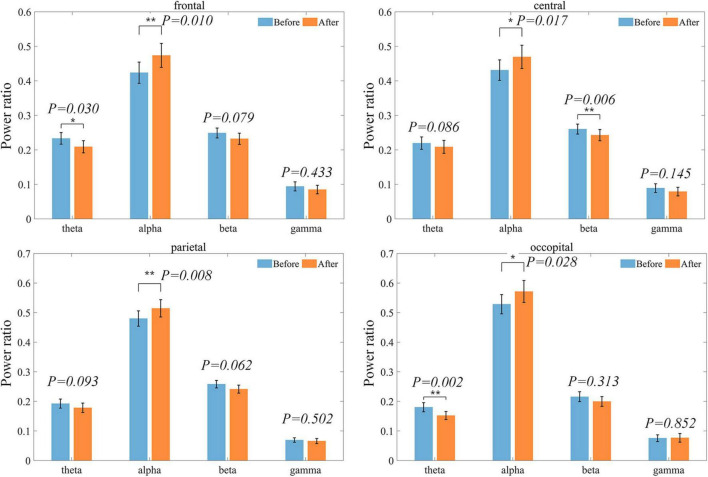
Distribution of power ratios in each band at each ROI before and after treatment. Bars indicating the mean and error bar representing the standard error. Power ratios are shown in blue before treatment and in orange after treatment.

The changes in each brain region are shown in Figure 5. Alpha band power ratio significantly increases in all four brain regions, while theta band power ratio was significantly decreased in the frontal and occipital regions. Beta band power was significantly reduced only in the central. Overall, in the ROIs where there was no significant difference but the difference between the mean before and after was large, there was also a tendency for the alpha band to increase and the other bands to decrease.

### 3.2 Difference between HC and LDH groups

**FIGURE 6:**
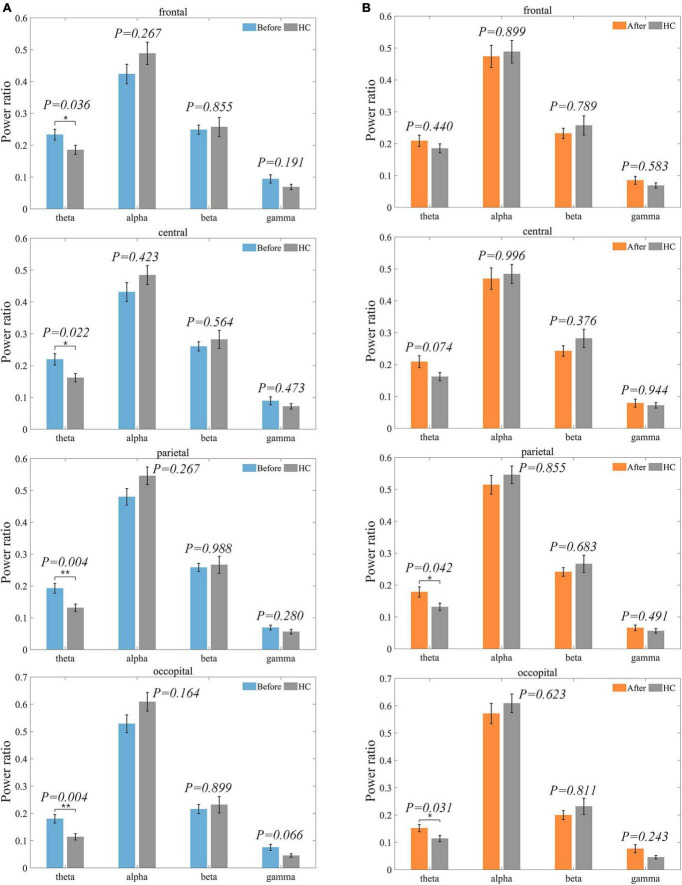
Distribution of power ratio of LDH group and HC group on each ROI before and after treatment. The power ratio of HC is shown in gray. *P*-value results of significance tests are labeled above each pair of bar graphs. **(A)** indicates the pre-treatment LDH group versus HC. **(B)** indicates the post-treatment LDH group versus HC.

The difference between the LDH group and HC was significant only in the theta frequency band. As shown in Figure 6A, the theta frequency band characteristics of the two groups were significantly different within all four ROIs before treatment, where the mean values of the LDH group compared to the HC group were higher in the parietal and occipital regions by 47.33 and 57.89%. The differences in frontal (25.95%) and central regions (35.80%) were slightly more minor. The differences between the two groups decreased after treatment (Figure 6B), with the results of the statistical test no longer significant in the frontal and central regions and the percentage of differences in the parietal and occipital regions decreasing to 35.88 and 33.33%. Hypothesis tests under other frequency bands and ROIs did not result in significance. Considering the before and after mean changes, the alpha power ratio was higher in healthy individuals than in the patient group, and the difference between the two groups became smaller after the treatment. The beta frequency band percentage was also higher in the HC than in the LDH group, but the gap between the two groups became larger after the treatment. The difference in gamma power ratio between the two groups became smaller after treatment. The detailed power ratio values of LDH and HC at each band and ROI are shown in Table 1.

**TABLE 1:** Mean and standard error of power ratios before and after treatment.

ROIs	Groups	Theta	Alpha	Beta	Gamma
Frontal	LDH before	0.233 ± 0.017	0.424 ± 0.031	0.249 ± 0.014	0.094 ± 0.013
	LDH after	0.209 ± 0.018	0.474 ± 0.035	0.232 ± 0.016	0.085 ± 0.012
	HC	0.185 ± 0.015	0.489 ± 0.036	0.257 ± 0.031	0.069 ± 0.009
Central	LDH before	0.220 ± 0.018	0.431 ± 0.030	0.260 ± 0.015	0.089 ± 0.012
	LDH after	0.209 ± 0.019	0.469 ± 0.034	0.243 ± 0.016	0.079 ± 0.013
	HC	0.162 ± 0.013	0.484 ± 0.031	0.282 ± 0.029	0.072 ± 0.009
Parietal	LDH before	0.193 ± 0.015	0.480 ± 0.026	0.258 ± 0.013	0.069 ± 0.008
	LDH after	0.178 ± 0.016	0.515 ± 0.029	0.241 ± 0.014	0.066 ± 0.009
	HC	0.131 ± 0.012	0.546 ± 0.029	0.266 ± 0.028	0.056 ± 0.008
Occipital	LDH before	0.180 ± 0.015	0.528 ± 0.032	0.216 ± 0.017	0.076 ± 0.011
	LDH after	0.152 ± 0.013	0.571 ± 0.037	0.200 ± 0.016	0.077 ± 0.015
	HC	0.114 ± 0.012	0.609 ± 0.035	0.232 ± 0.031	0.045 ± 0.006

### 3.3 Correlation of EEG with pain scales

**FIGURE 7:**
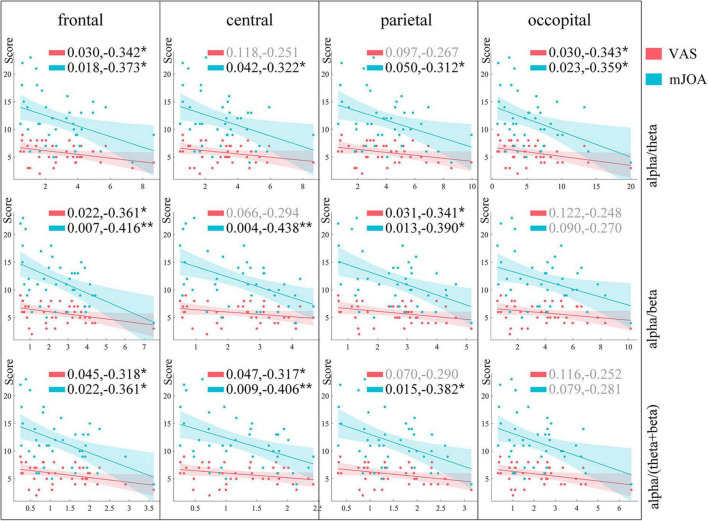
Results of correlation analysis between constructed features and pain scores. Rows represent the different features constructed, columns represent ROIs, and markers in the figure indicate *p*-values and correlation coefficients of correlation analysis results. Results of analyses with VAS scores and mJOA scores are shown in cyan and orange-red, respectively. *Represents different levels of *P*-value, *p < 0.05, **p < 0.01, ***p < 0.001.

In order to find a more appropriate EEG biomarker to quantify pain, we used theta, alpha, beta and gamma power ratio to do correlation analysis with the VAS scale as well as the mJOA, and the results of the correlation analysis are shown in Table 2. The EEG features correlated more strongly with the mJOA scale than VAS. The alpha band power ratio shows a significant negative correlation with mJOA scores at both frontal and central, and a slightly weaker negative correlation at parietal. Beta band power ratio is also correlated at frontal, central, and parietal, showing a positive correlation. Theta band power ratio shows a slightly weaker positive correlation at occipital with mJOA. There was a significant positive correlation with VAS scores only on beta band oscillations of frontal ROI. We continued to construct the three relative power features alpha/theta, alpha/beta, and alpha/(theta+beta) for use as correlation analyses. Figure 7 shows the results of the final analysis. The constructed alpha/beta feature performed optimally, showing weak negative correlations with VAS scores in both frontal (*R* = −0.361, *P* = 0.022) and parietal (*R* = −0.341, *P* = 0. 031), and more significant negative correlations with mJOA scores in the frontal (*R* = −0.416, *P* = 0.007), central (*R* = −0.438, *P* = 0.004), and parietal (*R* = −0.390, *P* = 0.013) regions. The other two features also showed a negative correlation trend. The power ratio of alpha/theta had a weaker negative correlation with VAS in the frontal (*R* = −0.342, *P* = 0.030), occipital (*R* = −0.343, *P* = 0.030), and with mJOA scores in the frontal (*R* = −0.373, *P* = 0.018), central (*R* = −0.322, *P* = 0.042), parietal (*R* = −0.312, *P* = 0.050), and occipital (*R* = −0.359, *P* = 0.023). Alpha/(theta+beta) power ratios likewise had a weaker negative correlation with VAS scores in frontal (*R* = −0.318, *P* = 0.045) and central (*R* = −0.317, *P* = 0.047), while negative correlation with mJOA scores in frontal (*R* = −0.361, *P* = 0.022), central (*R* = −0.406, *P* = 0.009), and parietal (*R* = −0.382, *P* = 0.015) was stronger compared to VAS scores.

**TABLE 2:** Results of the correlation analysis between EEG power ratio and pain scores at each ROI and each frequency band.

Scales	Bands	Frontal (*P*, *R*)	Central (*P*, *R*)	Parietal (*P*, *R*)	Occipital (*P*, *R*)
VAS	Theta	0.104, 0.261	0.242, 0.189	0.211, 0.202	0.065, 0.295
	Alpha	0.052, −0.310	0.096, −0.267	0.094, −0.268	0.197, −0.208
	Beta	0.040, 0.326*	0.202, 0.206	0.069, 0.291	0.257, 0.184
	Gamma	0.165, 0.224	0.278, 0.176	0.395, 0.138	0.532, 0.102
mJOA	Theta	0.053, 0.308	0.155, 0.229	0.105, 0.260	**0.017**, 0.376*
	Alpha	**0.010**, −0.401**	**0.015**, −0.381*	**0.027**, −0.350*	0.095, −0.267
	Beta	**0.007**, 0.417**	**0.013**, 0.388*	**0.014**, 0.386*	0.151, 0.231
	Gamma	0.256, 0.184	0.201, 0.207	0.388, 0.140	0.539, 0.100

*P* represents the *p*-value and *R* represents the correlation coefficient. Results with a *P*-value less than 0.05 are shown in bold in the table.

*p < 0.05,

**p < 0.01,

***p < 0.001.

## 4 Discussion

In this study, we recorded resting data in the closed-eye state of LDH patients before and after small needle knife, as well as in healthy subjects, and extracted the EEG power ratio features in four different frequency bands for four ROIs. The reduction of subjective pain perception after treatment was verified by the significant reduction of VAS and mJOA scores. The changes in EEG power ratio in the LDH group were further analyzed. It was found that small needle knife resulted in an increase in alpha power ratio in all brain ROIs, a significant decrease in theta band power ratio in the frontal and occipital regions, and a decrease in beta band in the central. However, the patient group had more low-frequency (theta) activity in all brain ROIs compared to HC, and the difference within theta frequency band was reduced after treatment.

Changes in the alpha frequency band were found to be the main EEG rhythm associated with pain in previous work (Schulz et al., 2015; Gram et al., 2017; Feng et al., 2021), and again the same conclusion was reached in our work. The pain scores of the patients were significantly lower after small needle knife, indicating a reduction in subjective pain perception. Alpha EEG increased significantly after treatment and was the dominant frequency band for EEG changes. In the correlation analysis between EEG and pain scores, alpha oscillations also showed the strongest negative correlation with pain scores compared to other frequency bands. A negative correlation of alpha oscillations and pain was likewise found in a study of lower back pain by correlation analysis of alpha power with pain scale scores (Feng et al., 2021). In previous work exploring biomarkers for the diagnosis of chronic pain intensity, a review summarized 25 relevant studies. Similarly alpha bands were found to be reported in more studies with a negative correlation with pain (Zebhauser et al., 2023). Similarly in a review of studies on evoked pain, a greater proportion of reports of negative correlation between alpha and pain were found (Zis et al., 2022).

Most of the previous related work has focused more on the specific manifestation of individual frequency bands in pain (Schulz et al., 2015; Bunk et al., 2018; Di Pietro et al., 2018; Feng et al., 2021). We found theta, alpha, and beta to be the dominant bands when comparing power ratios within the LDH group in this paper. And in the correlation analysis between individual bands and scale scores, we also found that the alpha showed a positive correlation trend with scores, and beta and theta showed a negative correlation trend. Therefore, we constructed 3 band ratio features [alpha/theta, alpha/beta, alpha/(theta+beta)] and hoped to find stronger correlations between pain by constructing these features to get more robust biomarkers. Band ratio features are a common analysis measure in cognitive and clinical neurological research, and are widely used in studies of fatigue (Jiang et al., 2024), learning and memory (Nokia et al., 2008), sleep (Reed et al., 2017), Alzheimer’s (Moretti et al., 2013), and autism (Wang et al., 2015). In a pain empathy study (Motoyama et al., 2017), subjects were observed to have suppression of alpha/beta in the frontocentral region and left parietooccipital region while watching a video of needling, and suppression of motor evoked potentials (MEPs) was observed with transcranial magnetic stimulation. The researchers concluded that alpha/beta is involved in the processing of somatic aspects of pain empathy and modulates inhibition of primary motor cortex excitability through somatosensory cortex (Motoyama et al., 2017). The alpha/beta band ratios we constructed were associated with negative correlations with the scale in the frontal, central, and parietal regions. The strongest correlations were found in the central region (the central region is located roughly above the somatosensory cortex), which may be related to the processing of the sensation of pain on the primary somatosensory cortex (Apkarian et al., 2005; Treede et al., 1999). However, band ratio is rarely used in pain studies, there is no more evidence to suggest a relationship between band ratio characteristics and the neural activity behind pain. The possibility of using band ratio features as biomarkers for pain assessment was explored in this study, which provides an idea of band compositing.

The theta band was the dominant band in the analysis of differences between HC and LDH patients. We found stronger theta oscillations on resting-state EEG in LDH patients compared to healthy individuals. In two other review articles on comparative studies of resting-state EEG data in healthy and chronic pain patients (Mussigmann et al., 2022; Zebhauser et al., 2023), the same is suggested in most studies showing more theta oscillations in pain patients compared to healthy individuals. Increased low-frequency (theta) activity in patients with LDH can be explained by the concept of thalamocortical dysrhythmia (TCD). The concept has been proposed as a general mechanism to explain the production of neuropathic pain and other neurological symptoms (Llinás et al., 1999; Sarnthein et al., 2006). TCD is based on a decrease in excitatory inputs or an increase in inhibitory inputs to thalamic neurons, resulting in the presence of persistent low-frequency thalamocortical resonance during the waking state (Llinás et al., 1999). In related neuropathic pain, this is reflected in an increase in low-frequency oscillations of the EEG in pain patients (Sarnthein et al., 2006). The TCD theory was similarly supported in an abdominal pain study (de Vries et al., 2013) and two studies of neuropathic pain after spinal cord injury (SCI) (Boord et al., 2008; Wydenkeller et al., 2009), which found an increase in low-frequency oscillations in patients. However, in patients with lower back pain (Schmidt et al., 2012), the phenomenon of TCD was not found in patients, and the researchers concluded that the intensity of the pain may influence the appearance of TCD.

Studies on pain EEG have differed in their choice of subjects, with one group examining changes in EEG activity in external stimulant-induced pain in healthy individuals (Schulz et al., 2015; Bunk et al., 2018; Wang et al., 2023),and another category of studies has controlled between patients with pain associated with neurological disorders and healthy individuals (Wydenkeller et al., 2009; de Vries et al., 2013; Xu B. et al., 2023). There is no consistent trend in the correlation between pain intensity and EEG features (Zebhauser et al., 2023). Chronic pain may disrupt multiple cortical circuits, thus affecting the processing of pain perception, and pain can be understood not only as an altered perceptual state but also as a result of changes in the way pain is processed by peripheral and central neurons (Kenefati et al., 2023). Previous work has found that chronic pain leads to maladaptive changes in the primary somatosensory cortex, the anterior cingulate cortex, dorsolateral prefrontal cortex, and the insular cortex (Apkarian et al., 2004; Mouraux and Iannetti, 2018; Chen, 2021). Kenefati et al. (2023) found that when subjected to mechanical stimulation, patients were more sensitive to the pain sensation of the stimulus compared to healthy individuals and that the medial orbitofrontal cortex was associated with this sensitivity. EEG patterns that healthy individuals exhibit in response to evoked pain may not be applicable to patients with chronic pain (Mouraux and Iannetti, 2018; Reckziegel et al., 2019; Mussigmann et al., 2022). In our study, we compared the healthy group with the patient group but also analyzed the differences at various pain levels within the patient group. Separating pain studies within patient groups from pain studies between groups allows for a more refined result and avoids errors in the results due to group crossover.

However, the two EEG acquisitions conducted before and after treatment were only separated by a relatively short period of time (about 20 min), which can only reflect the pattern of EEG to short-term pain changes. In the within-group comparison of LDH, alpha was the dominant band, with significant decreases in all four ROIs, theta was only in frontal and parietal, while beta was also significant in central. In the comparison between LDH and HC, then theta was the dominant band, with LDH showing stronger theta oscillations, with no significant differences in the alpha and beta bands. This also reminds us that the negative correlations between alpha and pain, and negative correlations between alpha/beta and subjective pain scores, are validated with the limitation that they may only be applicable within the group when there are short-term changes in pain.

The current work has some limitations. The current conclusions were drawn on patients with LDH and need to be expanded with studies in other clinical pain populations. Although our study underwent more careful signal noise processing and clinical pain assessment guidance to minimize individual differences. However, given the limited number of our trials, the results of interest may be subject to outliers. The present study did not finely delineate the duration of low back pain to validate the effect of pain duration on EEG patterns, which is also in need of further refinement in subsequent work. We only collected EEG for a short period of time before and after small needle knife therapy, which makes the results of the within-group analysis applicable to short-term pain changes and different from the results of the between-group comparisons. In the future, we hope to explore the pattern of within-group EEG changes under long-term interventions and verify whether the same pattern will be shown as between groups.

## 5 Conclusion

In this study, we found a reduction in pain scores in LDH after the small needle knife treatment, as well as a significant increase in the alpha power ratio of closed-eye resting-state EEG at four ROIs. In the frontal and central regions, alpha showed a negative correlation with subjective pain scores, beta showed a positive correlation, and the constructed alpha/beta feature showed the strongest negative correlation with subjective pain sensation. LDH patients had a greater theta power ratio than HC at all four ROIs, but the alpha power ratio, which is sensitive to the short-term reduction of subjective pain, did not show a significant increase between the LDH and HC groups. The use of alpha/beta as a biomarker should be restricted to the assessment of pain changes over short periods of time in patients with LDH. The present study preliminarily explored the possibility of resting-state EEG features as objective pain markers and their limitations.

## Data Availability

The original contributions presented in this study are included in this article/supplementary material, further inquiries can be directed to the corresponding authors.
